# Properly defining the targets of a transcription factor significantly improves the computational identification of cooperative transcription factor pairs in yeast

**DOI:** 10.1186/1471-2164-16-S12-S10

**Published:** 2015-12-09

**Authors:** Wei-Sheng Wu, Fu-Jou Lai

**Affiliations:** 1 Department of Electrical Engineering, National Cheng Kung University, Tainan, Taiwan

## Abstract

**Background:**

Transcriptional regulation of gene expression in eukaryotes is usually accomplished by cooperative transcription factors (TFs). Computational identification of cooperative TF pairs has become a hot research topic and many algorithms have been proposed in the literature. A typical algorithm for predicting cooperative TF pairs has two steps. (Step 1) Define the targets of each TF under study. (Step 2) Design a measure for calculating the cooperativity of a TF pair based on the targets of these two TFs. While different algorithms have distinct sophisticated cooperativity measures, the targets of a TF are usually defined using ChIP-chip data. However, there is an inherent weakness in using ChIP-chip data to define the targets of a TF. ChIP-chip analysis can only identify the binding targets of a TF but it cannot distinguish the true regulatory from the binding but non-regulatory targets of a TF.

**Results:**

This work is the first study which aims to investigate whether the performance of computational identification of cooperative TF pairs could be improved by using a more biologically relevant way to define the targets of a TF. For this purpose, we propose four simple algorithms, all of which consist of two steps. (Step 1) Define the targets of a TF using (i) ChIP-chip data in the first algorithm, (ii) TF binding data in the second algorithm, (iii) TF perturbation data in the third algorithm, and (iv) the intersection of TF binding and TF perturbation data in the fourth algorithm. Compared with the first three algorithms, the fourth algorithm uses a more biologically relevant way to define the targets of a TF. (Step 2) Measure the cooperativity of a TF pair by the statistical significance of the overlap of the targets of these two TFs using the hypergeometric test. By adopting four existing performance indices, we show that the fourth proposed algorithm (PA4) significantly out performs the other three proposed algorithms. This suggests that the computational identification of cooperative TF pairs is indeed improved when using a more biologically relevant way to define the targets of a TF. Strikingly, the prediction results of our simple PA4 are more biologically meaningful than those of the 12 existing sophisticated algorithms in the literature, all of which used ChIP-chip data to define the targets of a TF. This suggests that properly defining the targets of a TF may be more important than designing sophisticated cooperativity measures. In addition, our PA4 has the power to predict several experimentally validated cooperative TF pairs, which have not been successfully predicted by any existing algorithms in the literature.

**Conclusions:**

This study shows that the performance of computational identification of cooperative TF pairs could be improved by using a more biologically relevant way to define the targets of a TF. The main contribution of this study is not to propose another new algorithm but to provide a new thinking for the research of computational identification of cooperative TF pairs. Researchers should put more effort on properly defining the targets of a TF (i.e. Step 1) rather than totally focus on designing sophisticated cooperativity measures (i.e. Step 2). The lists of TF target genes, the Matlab codes and the prediction results of the four proposed algorithms could be downloaded from our companion website http://cosbi3.ee.ncku.edu.tw/TFI/

## Background

In eukaryotes, cooperativity among several transcription factors (TFs) is known to play an important role in transcriptional regulation. A relatively small number of cooperative TFs can set up very complex spatial and temporal patterns of gene expression. Knowing cooperative TFs is helpful for understanding the mechanisms of transcriptional regulation. Therefore, computational identification of cooperative TFs has become a hot research topic in modern biological research.

Many algorithms have been developed to identify cooperative TF pairs in yeast by integrating multiple high-throughput data sources such as gene expression data, ChIP-chip data, protein-protein interaction data, promoter sequence data, etc. [1-15]. The performance of an algorithm varies under different evaluation criteria [16]. A typical algorithm for predicting cooperative TF pairs has two steps. The first step is to define the targets of each TF under study and the second step is to design a measure for calculating the cooperativity of a TF pair based on the targets of these two TFs. While different algorithms propose distinct sophisticated cooperativity measures, the targets of a TF are usually defined using ChIP-chip data. However, there is an inherent weakness in using ChIP-chip data to define the targets of a TF. ChIP-chip analysis can only identify the binding targets of a TF but it cannot distinguish the true regulatory from the binding but non-regulatory targets of a TF [17].

This work is the first study which aims to investigate whether the performance of computational identification of cooperative TF pairs in yeast could be improved by using a more biologically relevant way to define the targets of a TF.

## Method

### Data sources

Four data sources were used in this study. First, 6017 TF-gene binding pairs for 168 TFs were retrieved from Harbison et al.'s ChIP-chip data with p-value less than 0.001 [2]. Each TF-gene binding pair was supported by the TF binding evidence from the high-throughput genome-wide ChIP-chip experiments in a single publication [2] showing that the TF binds to the promoter of the target gene. Second, 40761 TF-gene binding pairs for 170 TFs were retrieved from the TF binding data deposited in the YEASTRACT database [18]. Each TF-gene binding pair was supported by the TF binding evidence from the detailed gene by gene band-shift, foot-printing experiments or the high throughput genome-wide ChIP-chip experiments in different publications showing that the TF binds to the promoter of the target gene. Third, 165528 TF-gene regulation pairs for 294 TFs were retrieved from the TF perturbation data deposited in the YEASTRACT database [18]. Each TF-gene regulation pair was supported by the TF regulation evidence from the detailed gene by gene analysis or the genome-wide expression analysis in different publications showing that the perturbation (knockout or over-expression) of the TF-encoding gene causes a significant change in the expression of the target gene. Finally, we compiled 8609 TF-gene pairs for 151 TFs from the intersection of the TF binding and TF perturbation data deposited in the YEASTRACT database. Each TF-gene pair was supported by both the TF binding and TF regulation evidence. All the four data sources used in this study could be downloaded from our companion website.

### The four proposed algorithms

**Figure 1:**
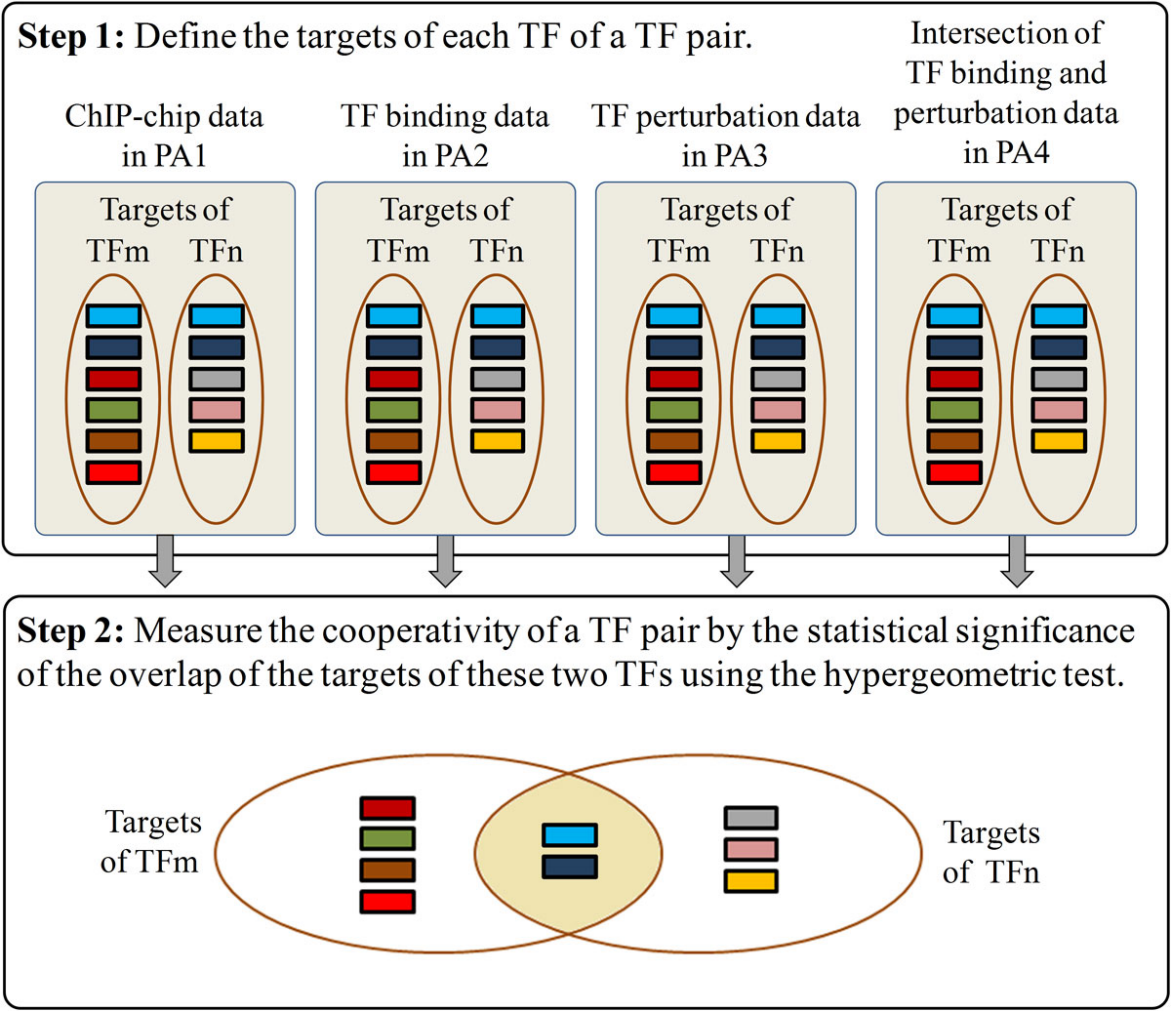
**An illustrative figure of the four proposed algorithms**. The four proposed algorithms all consist of two steps. Step 1: Define the targets of each TF of a TF pair. Step 2: Measure the cooperativity of a TF pair by the statistical significance of the overlap of the targets of these two TFs using the hypergeometric test. The four proposed algorithms all have the same Step 2 but have different Step 1. In Step1, the four proposed algorithms (denoted as PA1, PA2, PA3, and PA4) use different data sources to define the targets of a TF. PA1 uses ChIP-chip data, PA2 uses TF binding data, PA3 uses TF perturbation data, and PA4 uses the intersection of TF binding and TF perturbation data.

The four proposed algorithms all consist of two steps (see Figure 1). The first step is to define the targets of each yeast TF under study. The targets of a TF are defined using (i) ChIP-chip data in the first algorithm (just like many existing algorithms in the literature), (ii) TF binding data in the second algorithm, (iii) TF perturbation data in the third algorithm, and (iv) the intersection of TF binding and TF perturbation data in the fourth algorithm. Compared with the first three algorithms, the fourth algorithm uses a more biologically relevant way to define the targets of a TF since all the targets are bound and regulated by this TF supported by both the TF binding and TF regulation evidence.

The second step of the proposed algorithms is to design a measure for calculating the cooperativity of a TF pair based on the targets of these two TFs. Since the biological role of a cooperative TF pair is to co-regulate the expression of a set of genes, the number of the common targets of a cooperative TF pair should be significantly higher than that of a random TF pair. In other words, the overlap of the targets of a cooperative TF pair should be significantly higher than that of a random TF pair [19]. Therefore, the proposed algorithms measure the cooperativity of a TF pair based on the statistical significance of the overlap of the targets of these two TFs. The statistical significance is computed using the hypergeometric test [20] as follows:

(1) $$p\text{\_value}=P\left(i\geq m\right)=\overset{\text{min}\left(N_{1},N_{2}\right)}{\underset{i=m}{\sum }}\frac{\left(\begin{array}{c} N_{1}\\ i \end{array}\right)\left(\begin{array}{c} G-N_{1}\\ N_{2}-i \end{array}\right)}{\left(\begin{array}{c} G\\ N_{2} \end{array}\right)}$$

where *G *= 6575 is the number of genes in the yeast genome, *N*_1 _is the number of the targets of the first TF, *N*_2 _is the number of the targets of the second TF, *m *is the number of common targets of these two TFs. In summary, the smaller the p-value, the higher the chance that a TF pair has cooperativity.

Note that in Step 1, the targets of 168, 170, 294, and 151 TFs can be defined for the first, second, third, and fourth proposed algorithm, respectively (see Data sources section for details). Therefore, in Step 2, the cooperativity of 14028 (168*167/2), 14365 (170*169/2), 43071 (294*293/2), and 11325 (151*150/2) TF pairs can be computed for the first, second, third, and fourth proposed algorithm, respectively. For each algorithm, these TF pairs were then sorted by their p-values, where the top one TF pair has the smallest p-value and therefore is the most statistically significant cooperative TF pair. For example, the output of the fourth algorithm is a ranked list of 11325 TF pairs. The detailed descriptions of the four proposed algorithms could be found in Additional file 1.

### Four performance comparison indices

We adopt four existing performance comparison indices from the literature to evaluate the performance of an algorithm in identifying cooperative TF pairs. These four indices are introduced in the following subsections.

#### Performance index 1: The statistical significance of the overlap with the benchmark set

Yang et al. [13] compiled a benchmark set of 27 known cooperative TF pairs from the MIPS transcription complex catalogues [21]. Then they computed the statistical significance of the overlap of the set of the predicted cooperative TF pairs (PCTFPs) from an algorithm with the benchmark set to evaluate the performance of an algorithm. The statistical significance (p-value) is calculated using the Fisher exact test. The larger the -log(p-value), the greater the statistical significance. Therefore, the larger the -log(p-value), the better the performance of an algorithm.

#### Performance index 2: The similarity of protein-protein interaction (PPI) partners between the two TFs of each PCTFP

The similarity of PPI partners between two TFs may suggest that they participate in the same regulatory mechanism. This rationale has been used in previous studies [15,16] to evaluate the biological plausibility of a PCTFP. The physical PPI data were downloaded from the BioGRID database [22]. The PPI partners similarity score of a TF pair, denoted as -log(p-value), is calculated using Equation (1). Note that *G *= 6575 is the number of genes in the yeast genome, *N*_1 _is the number of proteins which have physical PPI with the first TF, *N*_2 _is the number of proteins which have physical PPI with the second TF, and *m *is the number of common PPI partners of these two TFs. Here we use the average of the PPI partners similarity scores of all PCTFPs from an algorithm to evaluate the performance of an algorithm. The larger the average, the better the performance of an algorithm.

#### Performance index 3: The shortest path length of two TFs in the physical PPI network

Aguilar and Oliva [23] observed that a cooperative TF pair has a shorter path length in the physical PPI network than random expectation. The physical PPI network is constructed using the physical PPI data retrieved from the BioGRID database [22]. Here we use the average of the reciprocals of the shortest path lengths of all PCTFPs from an algorithm to evaluate the performance of an algorithm. The larger the average, the better the performance of an algorithm.

#### Performance index 4: The functional similarity of two TFs

Since a cooperative TF pair co-regulates the expression of a set of genes, they should have similar functions. Functional similarity has been used in several previous studies [10,15,16] to evaluate the biological plausibility of a PCTFP. The functional similarity score of a TF pair, which is calculated based on their GO semantic similarity, was retrieved from Yang et al.'s study [24]. Here we use the average of the functional similarity scores of all PCTFPs from an algorithm to evaluate the performance of an algorithm. The larger the average, the better the performance of an algorithm.

## Results and discussion

By adopting four existing performance comparison indices from the literature, we have the following discoveries.

### The performance of computational identification of cooperative TF pairs could be improved by using a more biologically relevant way to define the targets of a TF

In this study, the four proposed algorithms (denoted as PA1, PA2, PA3, and PA4) use different ways to define the targets of a TF. The targets of a TF defined by PA1 and PA2 (using ChIP-chip data and TF binding data, respectively) are bound but not necessarily are regulated by this TF. The targets of a TF defined by PA3 (using TF perturbation data) are regulated but not necessarily are bound by this TF. The targets of a TF defined by PA4 (using the intersection of the TF binding and TF perturbation data) are bound and regulated by this TF. Therefore, the targets of a TF defined by PA4 are more biologically relevant than those defined by the other three algorithms.

**Figure 2:**
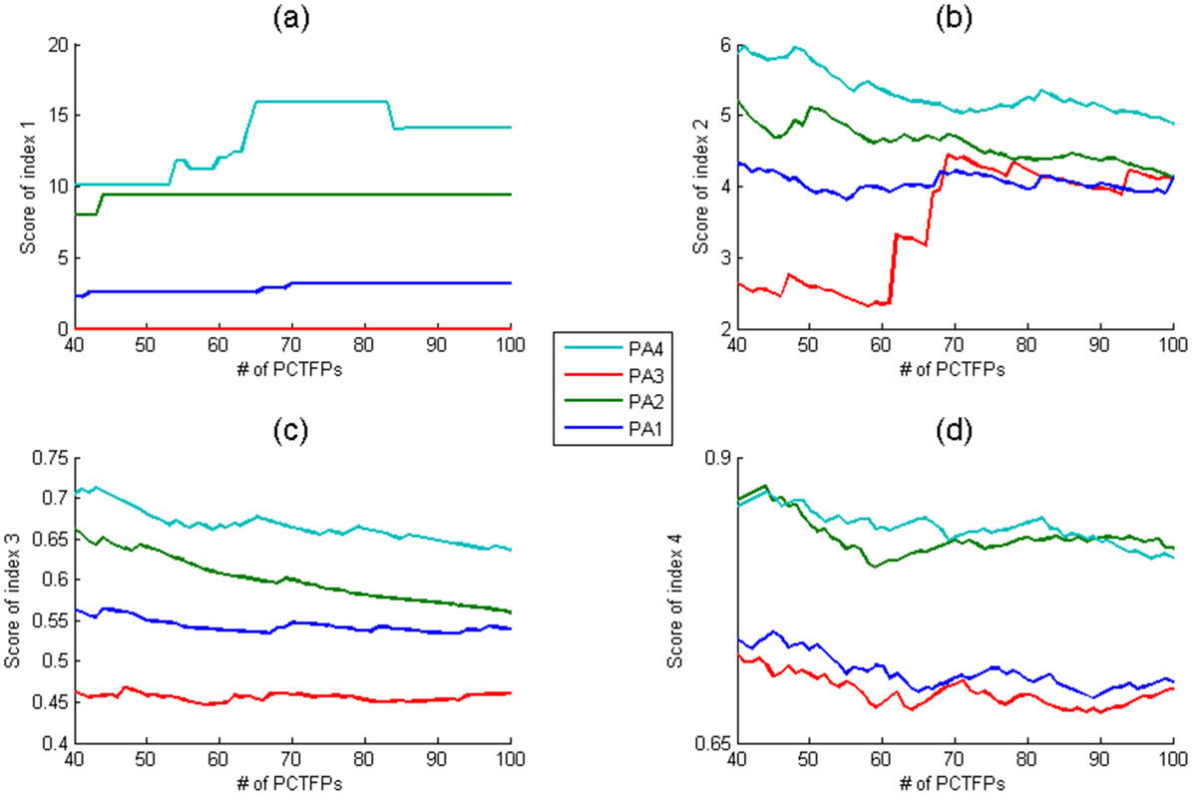
**Performance comparison of PA1, PA2, PA3 and PA4 using four existing performance indices**. PAi means i-th proposed algorithm. The x-axis is the number of the predicted cooperative TF pairs (PCTFPs) being reported. For example, 50 means the top 50 PCTFPs in the ranked list of TF pairs generated by an algorithm. The y-axis is defined as follows. (a) The score of index 1 is the negative logarithm of the statistical significance (p-value) of the overlap of the PCTFPs from an algorithm with the benchmark set. The larger the -log(p-value), the better the performance of an algorithm. (b) The score of index 2 is the average of the PPI partners similarity scores of all PCTFPs from an algorithm. The larger the average, the better the performance of an algorithm. (c) The score of index 3 is the average of the reciprocals of the shortest path lengths in the physical PPI network of all PCTFPs from an algorithm. The larger the average, the better the performance of an algorithm. (d) The score of index 4 is the average of the functional similarity scores of all PCTFPs from an algorithm. The larger the average, the better the performance of an algorithm.

Here we compare the performance of the four proposed algorithms using the four existing performance indices in the literature. Figure 2 shows that PA4 outperforms the other three algorithms in almost all the indices and almost all the chosen numbers of the top PCTFPs being reported. Our analyses suggest that using a more biologically relevant way to define the targets of a TF indeed helps identify cooperative TF pairs.

### Performance comparison of the fourth proposed algorithm (PA4) with 12 existing sophisticated algorithms in the literature

**Figure 3:**
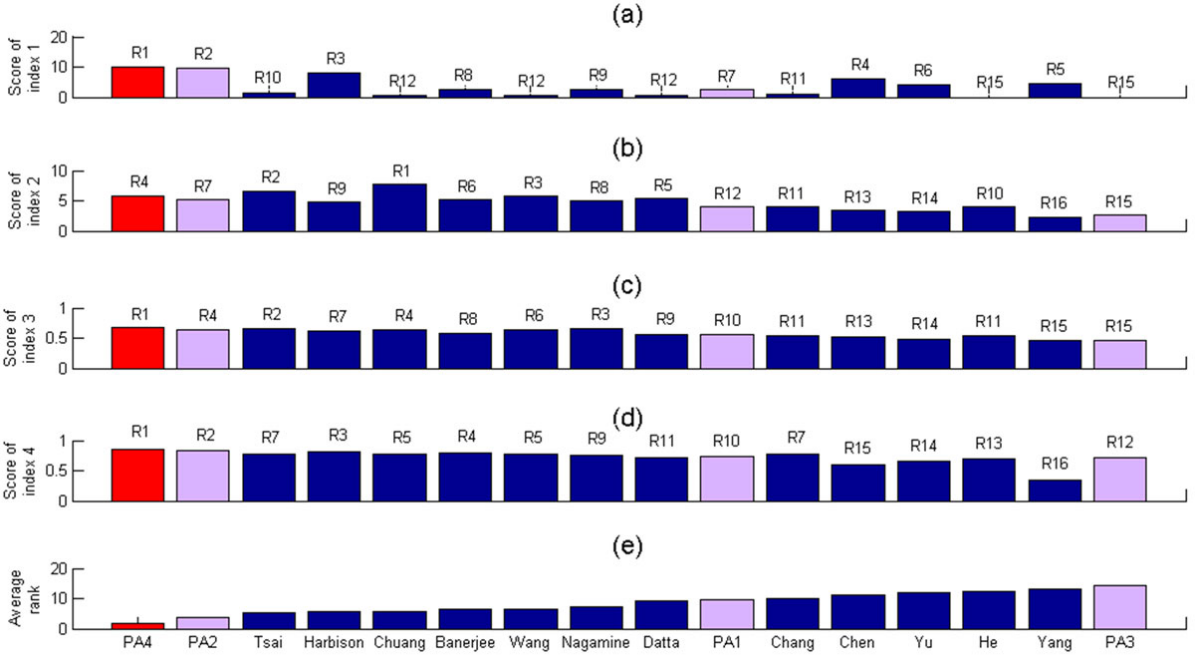
**Performance comparison of the four proposed algorithms and 12 existing algorithms in the literature**. Performance comparison of our PA1, PA2, PA3 and PA4 and 12 existing algorithms using four existing performance indices. The performance comparison results using (a) index 1, (b) index 2, (c) index 3 and (d) index 4 are shown, where Rj means that the algorithm is ranked j among the 16 compared algorithms. For example, our PA4 is ranked first (R1) using the performance index 1 since our PA4 has the largest score calculated using the performance index 1. (e) The average rank is used to give the overall performance of an algorithm under four different performance indices. The average rank of an algorithm is the average of the ranks of an algorithm under four performance indices. For example, the average rank of our PA4 is 1.75 = (1+4+1+1)/4 and the average rank of Harbison et al.'s algorithm is 5.5 = (3+9+7+3)/4. The smaller the average rank, the better the performance of an algorithm. It can be seen that our PA4 has the smallest average rank. Therefore, the overall performance of our PA4 is the best among all the 16 compared algorithms.

Here we compare the performances of our PA4 and 12 existing algorithms [1-7,9-11,13,14] in the literature. The differences between our PA4 and these 12 existing algorithms are as follows. First, our PA4 integrates TF binding and TF perturbation data but the 12 existing algorithms all use ChIP-chip data to define the targets of a TF. Second, the cooperativity measures proposed by these 12 existing algorithms are much more sophisticated than that of our PA4. In order to conduct the performance comparison, we consider the top 50 TF pairs with the hypergeometric test p-values less than 2 × 10^-15 ^as the PCTFPs of our PA4. Reporting the top 50 TF pairs seems reasonable because the number of the PCTFPs of most existing algorithms [1,3,5,6,8,9,15] falls between 20 and 60. For completeness of the comparison, we also consider the top 50 PCTFPs from PA1, PA2 and PA3 as their predictions. Figure 3 shows that PA4 is the best performing algorithm, which has the smallest average rank, among the 16 compared algorithms. Moreover, the performance of PA2, PA1 and PA3 ranks 2, 10, and 16, respectively, among the 16 compared algorithms. Note that the comparison is on the PCTFPs but not on the algorithms themselves since different algorithms cannot be compared fairly due to using different kinds of data sources. In summary, our finding suggests that properly defining the targets of a TF may be more important than designing sophisticated cooperativity measures.

### The fourth proposed algorithm (PA4) is robust against different p-value thresholds for determining the PCTFPs

**Figure 4:**
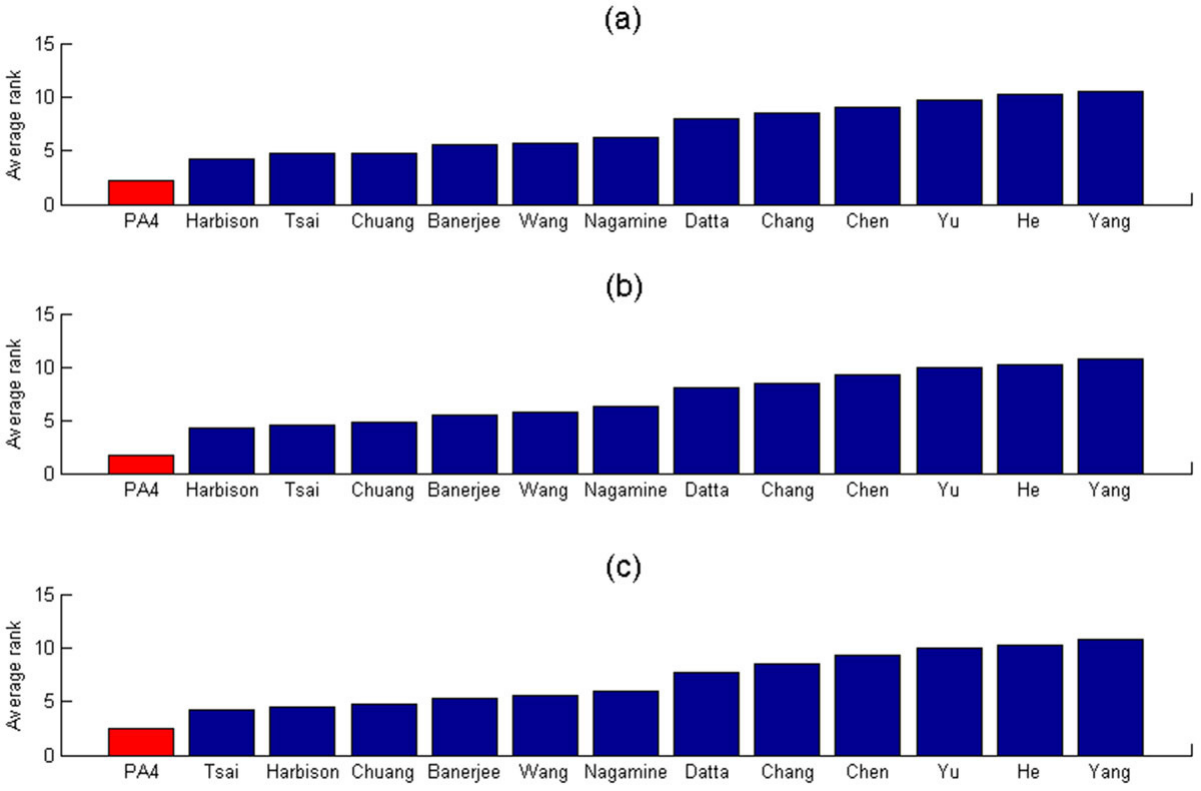
**Robustness analysis of the fourth proposed algorithm (PA4)**. The average rank of our PA4 when using (a) 10^-25^, (b) 10^-20 ^and (c) 10^-10 ^as the p-value thresholds for determining the number of the reported predicted cooperative TF pairs (PCTFPs), which is (a) 22, (b) 33 and (c) 88, respectively. It can be seen that the PCTFPs from our PA4 are always more biologically meaningful than those from the 12 existing algorithms since our PA4 always has the smallest average rank no matter which p-value threshold is used. This suggests that our PA4 is robust against different p-value thresholds.

In the last subsection, our PA4 set 2 × 10^-15 ^as the p-value threshold of the hypergeometric test and reported 50 PCTFPs whose p-values are less than the threshold. We then showed that the PCTFPs from our PA4 are more biologically meaningful than those from the 12 existing algorithms in the literature. To check the robustness of our PA4 against different p-value thresholds, here we evaluate the performance of our PA4 using three other different p-value thresholds (10^-25^, 10^-20 ^and 10^-10^), which reports 22, 33, 88 PCTFPs, respectively. Figure 4 shows that no matter which p-value threshold is used, our PA4 always has a smaller average rank than do the 12 existing algorithms in the literature. This suggests that our PA4 is indeed robust against different p-value thresholds.

### The fourth proposed algorithm (PA4) predicts nine unique cooperative TF pairs

In this study, our PA4 reports 50 PCTFPs (see Additional file 2). Among them, nine pairs are unique PCTFPs, which have not been predicted by any existing algorithms (see Table 1). Strikingly, four of the nine unique pairs are experimentally validated cooperative TF pairs. For the other five unique pairs, the two TFs of each pair both participate in the same biological process, suggesting that they may co-regulate genes involved in that specific biological process.

**Table 1:** Nine unique PCTFPs (among the 50 PCTFPs) which are predicted by the fourth proposed algorithm but not by any existing algorithms

Rank	PCTFP	Experimental evidence	The biological process in which both TFs are involved
1	**Ifh1-Sfp1**	Jorgensen et al. [25]	Regulation of ribosomal protein gene transcription

2	**Ifh1-Rap1**	Wade et al. [26]	Regulation of ribosomal protein gene transcription

16	Msn2-Ste12		Stress response

17	Msn2-Tec1		Stress response

20	**Ifh1-Fhl1**	Schawalder et al. [27]	Regulation of ribosomal protein gene transcription

30	Msn2-Pdr1		Stress response

42	Sok2-Ste12		Pseudohyphal growth

44	**Rap1-Tup1**	Roth [28]	Chromatin-mediated transcription regulation

46	Msn2-Rap1		Stress response

Bold-faced PCTFPs are experimentally validated cooperative TF pairs.

Two PCTFPs Ifh1-Sfp1 and Ifh1-Rap1 are noteworthy. These two pairs are the top two most statistically significant (ranked first and second) cooperative TF pairs predicted by our PA4 and they have not been predicted by any existing algorithm. Remarkably, these two PCTFPs have been experimentally validated in the literature. It is known that Sfp1 influences the nuclear localization of Ifh1, which binds to ribosomal protein (RP) gene promoters. The absence of Sfp1 causes Ifh1 to localize to nucleolar regions, thus reducing RP gene transcription [25]. In addition, the RP gene promoter is characterized by upstream binding of the general TF Rap1 followed by the RP gene specific TF Ifh1 via the forkhead-associated domain of Fhl1 [26].

The fact that only our PA4 but no existing algorithms can predict the four experimentally validated cooperative TF pairs (Ifh1-Sfp1, Ifh1-Rap1, Ifh1-Fhl1, and Rap1-Tup1) convincingly demonstrates the usefulness of our PA4.

## Conclusions

In this study, we investigated whether the performance of computational identification of cooperative TF pairs could be improved by using a more biologically relevant way to define the targets of a TF. We developed a simple algorithm (i.e. the fourth proposed algorithm PA4) which integrates TF binding data and TF perturbation data to define the biologically plausible targets of a TF. Our PA4 predicts nine unique PCTFPs, which have not been predicted by any existing algorithms. Remarkably, four of the nine unique pairs are experimentally validated cooperative TF pairs, convincingly demonstrating the usefulness of our PA4. Moreover, by adopting four existing performance comparison indices from the literature, we have two discoveries. First, the performance of computational identification of cooperative TF pairs is improved when integrating TF binding and TF perturbation data instead of using ChIP-chip data alone, TF binding data alone or TF perturbation data alone to define the targets of a TF. This suggests that using a more biologically relevant way to define the targets of a TF indeed helps identify cooperative TF pairs. Second, the cooperative TF pairs predicted by our simple PA4 are more biologically relevant than those predicted by the 12 existing sophisticated algorithms. This suggests that properly defining the targets of a TF may be more important than designing sophisticated cooperativity measures. In conclusion, our study shows that how to define the targets of a TF in a more biologically relevant way is critical for successful identification of cooperative TF pairs. Researchers should put more effort on properly defining the targets of a TF rather than totally focus on designing sophisticated cooperativity measures.

## Competing interests

The authors declare that they have no competing interests.

## Authors' contributions

WSW conceived the research topic, developed the method and wrote the manuscript. FJL did all the simulations and prepared all the figures. Both authors read, edited and approved the final manuscript.

## Supplementary Material

Additional file 1 **The detailed descriptions of the four proposed algorithms**. Click here for file

Additional file 2 **The 50 PCTFPs from the fourth proposed algorithm**. Click here for file

## Declarations

The publication of this paper was funded by National Cheng Kung University and Ministry of Science and Technology of Taiwan MOST-103-2221-E-006-174-MY2.

This article has been published as part of *BMC Genomics *Volume 16 Supplement 12, 2015: Joint 26th Genome Informatics Workshop and 14th International Conference on Bioinformatics: Genomics. The full contents of the supplement are available online at http://www.biomedcentral.com/bmcgenomics/supplements/16/S12.
