# Supplementary material for: Properly defining the targets of a transcription factor significantly improves the computational identification of cooperative transcription factor pairs in yeast
Source: BMC Genomics. 2015 Dec 9;16(Suppl 12):S10. doi: 10.1186/1471-2164-16-S12-S10 (PMC4682405; doi:10.1186/1471-2164-16-S12-S10)
Supplement: Additional file 1 — The detailed descriptions of the four proposed algorithms. [file 1471-2164-16-S12-S10-S1.pdf]

The detailed description of the first proposed algorithm (PA1)

Step 1: Define the targets of 168 TFs using ChIP-chip data

The 6017 TF-gene binding pairs for 168 TFs were retrieved from Harbison et al.'s ChIP-chip data with p-value less than 0.001 [1]. Each TF-gene binding pair was supported by the TF binding evidence from the high-throughput genome-wide ChIP-chip experiments in a single publication [1] showing that the TF binds to the promoter of the target gene.

Step 2: Measure the cooperativity of each of the 14028 (168*167/2) TF pairs based on the targets of the two TFs in a TF pair

Since the biological role of a cooperative TF pair is to co-regulate the expression of a set of genes, the number of the common targets of a cooperative TF pair should be significantly higher than that of a random TF pair. In other words, the overlap of the targets of a cooperative TF pair should be significantly higher than that of a random TF pair [2]. Therefore, the proposed algorithms measure the cooperativity of a TF pair based on the statistical significance of the overlap of the targets of these two TFs. The statistical significance is computed using the hypergeometric test [3] as follows:

$$p\_\text{value} = P(i \geq m) = \sum_{i=m}^{\min(N_1,N_2)} \frac{\binom{N_1}{i}\binom{G-N_1}{N_2-i}}{\binom{G}{N_2}}$$

where $G = 6575$ is the number of genes in the yeast genome, N_1 is the number of the targets of the first TF, N_2 is the number of the targets of the second TF, m is the number of common targets of these two TFs. In summary, the smaller the p-value, the higher the chance that a TF pair has cooperativity.

After running Step 2 for each of the 14028 TF pairs, these 14028 TF pairs were then sorted by their p-values, where the top one TF pair has the smallest p-value and therefore is the most statistically significant cooperative TF pair. Finally, the output of the proposed algorithm is a ranked list of 14028 TF pairs.

References

1. Harbison CT, Gordon DB, Lee TI, Rinaldi NJ, Macisaac KD, Danford TW, Hannett NM, Tagne JB, Reynolds DB, Yoo J, Jennings EG, Zeitlinger J, Pokholok DK, Kellis M, Rolfe PA, Takusagawa KT, Lander ES, Gifford DK,

Fraenkel E, Young RA: **Transcriptional regulatory code of a eukaryotic genome.** *Nature* 2004, **431**:99-104.
2. Garten Y, Kaplan S, Pilpel Y: **Extraction of transcription regulatory signals from genome-wide DNA-protein interaction data.** *Nucleic Acids Res* 2005, **33(2)**:605-615.
3. Wu WS, Li WH: **Systematic identification of yeast cell cycle transcription factors using multiple data sources.** *BMC Bioinformatics* 2008, **9:**522.

The detailed description of the second proposed algorithm (PA2)

Step 1: Define the targets of 170 TFs using TF binding data

The 40761 TF-gene binding pairs for 170 TFs were retrieved from the TF binding data deposited in the YEASTRACT database [1]. Each TF-gene binding pair was supported by the TF binding evidence from the detailed gene by gene band-shift, foot-printing experiments or the high throughput genome-wide ChIP-chip experiments in different publications showing that the TF binds to the promoter of the target gene.

Step 2: Measure the cooperativity of each of the 14365 (170*169/2) TF pairs based on the targets of the two TFs in a TF pair

Since the biological role of a cooperative TF pair is to co-regulate the expression of a set of genes, the number of the common targets of a cooperative TF pair should be significantly higher than that of a random TF pair. In other words, the overlap of the targets of a cooperative TF pair should be significantly higher than that of a random TF pair [2]. Therefore, the proposed algorithms measure the cooperativity of a TF pair based on the statistical significance of the overlap of the targets of these two TFs. The statistical significance is computed using the hypergeometric test [3] as follows:

$$p\_\text{value} = P(i \geq m) = \sum_{i=m}^{\min(N_1,N_2)} \frac{\binom{N_1}{i}\binom{G-N_1}{N_2-i}}{\binom{G}{N_2}}$$

where $G = 6575$ is the number of genes in the yeast genome, N_1 is the number of the targets of the first TF, N_2 is the number of the targets of the second TF, m is the number of common targets of these two TFs. In summary, the smaller the p-value, the higher the chance that a TF pair has cooperativity.

After running Step 2 for each of the 14365 TF pairs, these 14365 TF pairs were then sorted by their p-values, where the top one TF pair has the smallest p-value and therefore is the most statistically significant cooperative TF pair. Finally, the output of the proposed algorithm is a ranked list of 14365 TF pairs.

References

1. Abdulrehman D, Monteiro PT, Teixeira MC, Mira NP, Lourenço AB, dos Santos SC, Cabrito TR, Francisco AP, Madeira SC, Aires RS, Oliveira AL, Sá-Correia I, Freitas AT: **YEASTRACT: providing a programmatic access to curated transcriptional regulatory associations in Saccharomyces cerevisiae through a web services interface.** *Nucleic Acids Res* 2011, **39**:D136-D140.
2. Garten Y, Kaplan S, Pilpel Y: **Extraction of transcription regulatory signals from genome-wide DNA-protein interaction data.** *Nucleic Acids Res* 2005, **33(2)**:605-615.
3. Wu WS, Li WH: **Systematic identification of yeast cell cycle transcription factors using multiple data sources.** *BMC Bioinformatics* 2008, **9:**522.

The detailed description of the third proposed algorithm (PA3)

Step 1: Define the targets of 294 TFs using TF perturbation data

The 165528 TF-gene regulation pairs for 294 TFs were retrieved from the TF perturbation data deposited in the YEASTRACT database [1]. Each TF-gene regulation pair was supported by the TF regulation evidence from the detailed gene by gene analysis or the genome-wide expression analysis in different publications showing that the perturbation (knockout or over-expression) of the TF-encoding gene causes a significant change in the expression of the target gene.

Step 2: Measure the cooperativity of each of the 43071 (294*293/2) TF pairs based on the targets of the two TFs in a TF pair

Since the biological role of a cooperative TF pair is to co-regulate the expression of a set of genes, the number of the common targets of a cooperative TF pair should be significantly higher than that of a random TF pair. In other words, the overlap of the targets of a cooperative TF pair should be significantly higher than that of a random TF pair [2]. Therefore, the proposed algorithms measure the cooperativity of a TF pair

based on the statistical significance of the overlap of the targets of these two TFs. The statistical significance is computed using the hypergeometric test [3] as follows:

$$p\_value = P(i \geq m) = \sum_{i=m}^{\min(N_1,N_2)} \frac{\binom{N_1}{i}\binom{G-N_1}{N_2-i}}{\binom{G}{N_2}}$$

where $G = 6575$ is the number of genes in the yeast genome, N_1 is the number of the targets of the first TF, N_2 is the number of the targets of the second TF, m is the number of common targets of these two TFs. In summary, the smaller the p-value, the higher the chance that a TF pair has cooperativity.

After running Step 2 for each of the 43071 TF pairs, these 43071 TF pairs were then sorted by their p-values, where the top one TF pair has the smallest p-value and therefore is the most statistically significant cooperative TF pair. Finally, the output of the proposed algorithm is a ranked list of 43071 TF pairs.

References

1. Abdulrehman D, Monteiro PT, Teixeira MC, Mira NP, Lourenço AB, dos Santos SC, Cabrito TR, Francisco AP, Madeira SC, Aires RS, Oliveira AL, Sá-Correia I, Freitas AT: **YEASTRACT: providing a programmatic access to curated transcriptional regulatory associations in Saccharomyces cerevisiae through a web services interface.** *Nucleic Acids Res* 2011, **39**:D136-D140.
2. Garten Y, Kaplan S, Pilpel Y: **Extraction of transcription regulatory signals from genome-wide DNA-protein interaction data.** *Nucleic Acids Res* 2005, **33(2)**:605-615.
3. Wu WS, Li WH: **Systematic identification of yeast cell cycle transcription factors using multiple data sources.** *BMC Bioinformatics* 2008, **9:**522.

The detailed description of the fourth proposed algorithm (PA4)

Step 1: Define the targets of 151 TFs using the intersection of TF binding and TF perturbation data

We compiled 8609 TF-gene pairs for 151 TFs from the intersection of the TF binding and TF perturbation data deposited in the YEASTRACT database [1]. Each TF-gene

pair was supported by both the TF binding and TF regulation evidence.

Step 2: Measure the cooperativity of each of the 11325 (151*150/2) TF pairs based on the targets of the two TFs in a TF pair

Since the biological role of a cooperative TF pair is to co-regulate the expression of a set of genes, the number of the common targets of a cooperative TF pair should be significantly higher than that of a random TF pair. In other words, the overlap of the targets of a cooperative TF pair should be significantly higher than that of a random TF pair [2]. Therefore, the proposed algorithms measure the cooperativity of a TF pair based on the statistical significance of the overlap of the targets of these two TFs. The statistical significance is computed using the hypergeometric test [3] as follows:

$$p\_\text{value} = P(i \geq m) = \sum_{i=m}^{\min(N_1,N_2)} \frac{\binom{N_1}{i}\binom{G-N_1}{N_2-i}}{\binom{G}{N_2}}$$

where $G = 6575$ is the number of genes in the yeast genome, N_1 is the number of the targets of the first TF, N_2 is the number of the targets of the second TF, m is the number of common targets of these two TFs. In summary, the smaller the p-value, the higher the chance that a TF pair has cooperativity.

After running Step 2 for each of the 11325 TF pairs, these 11325 TF pairs were then sorted by their p-values, where the top one TF pair has the smallest p-value and therefore is the most statistically significant cooperative TF pair. Finally, the output of the proposed algorithm is a ranked list of 11325 TF pairs.

References

1. Abdulrehman D, Monteiro PT, Teixeira MC, Mira NP, Lourenço AB, dos Santos SC, Cabrito TR, Francisco AP, Madeira SC, Aires RS, Oliveira AL, Sá-Correia I, Freitas AT: **YEASTRACT: providing a programmatic access to curated transcriptional regulatory associations in Saccharomyces cerevisiae through a web services interface.** *Nucleic Acids Res* 2011, **39**:D136-D140.
2. Garten Y, Kaplan S, Pilpel Y: **Extraction of transcription regulatory signals from genome-wide DNA-protein interaction data.** *Nucleic Acids Res* 2005, **33(2)**:605-615.
3. Wu WS, Li WH: **Systematic identification of yeast cell cycle transcription factors using multiple data sources.** *BMC Bioinformatics* 2008, **9:**522.
